# Contrasting population genetic structures of congeneric coastal flounders associated with different early life histories

**DOI:** 10.1111/jfb.70461

**Published:** 2026-05-21

**Authors:** Yuki Yamamoto, Hiroyuki Togashi, Yutaka Kurita, Satoshi Katayama, Yoshiaki Akaba, Mitsuhiro Ishii, Tatsuya Isshiki, Minoru Ikeda

**Affiliations:** ^1^ Onagawa Field Center, Graduate School of Agricultural Science Tohoku University Onagawa Japan; ^2^ Fisheries Resources Institute Japan Fisheries Research and Education Agency Yokohama Japan; ^3^ Fisheries Resources Institute Japan Fisheries Research and Education Agency Shiogama Japan; ^4^ Graduate School of Agricultural Science Tohoku University Sendai Japan; ^5^ Tohoku University & JAMSTEC Advanced Institute for Marine Ecosystem Change (WPI‐AIMEC) Sendai Japan; ^6^ Chiba Prefectural Fisheries Research Center Chiba Japan; ^7^ Kanagawa Prefectural Fisheries Technology Center Miura Japan

**Keywords:** demographic history, egg trait, fisheries management, mitochondrial DNA, planktonic dispersal

## Abstract

Marine fish species are likely to exhibit little genetic differentiation among populations due to their high dispersal potential during early life stages and migratory nature. However, recent studies have increasingly reported intraspecific genetic differentiation resulting from species‐specific ecological traits, environmental factors, and paleogeographic changes associated with glacial–interglacial cycles. Egg traits are considered to affect the level of genetic differentiation among populations; however, little is known about the specific differences in population genetic structure that result from variations in egg traits between closely related species. Using mitochondrial DNA sequences from the NADH dehydrogenase subunit 2 and cytochrome *b* genes, we compared population genetic structures of two congeneric coastal flounders around the Japanese Archipelago, yellow striped flounder *Pseudopleuronectes herzensteini* and marbled flounder *P. yokohamae*, which exhibit contrasting early life‐history traits related to dispersal, including egg traits. No genetic differentiation was observed among sampling locations of *P. herzensteini* (global *F*_ST_ = −0.002), which spawns pelagic eggs and has a longer planktonic larval duration than *P. yokohamae*. Historical demographic analyses suggest that *P. herzensteini* has maintained a large population size over time, with extensive gene flow across the sampling area. In contrast, six genetic groups were identified in the adhesive demersal egg spawner *P. yokohamae*, with some genetic barriers to gene flow among them (global *F*_ST_ = 0.171). Distinctive population demographic histories were inferred for each group, suggesting that paleogeographic changes during the Late Pleistocene greatly influenced the local population formation. These findings indicate that the highly sedentary characteristics in early life stages of *P. yokohamae* lead to greater population divergence and distinct demographic histories. Such findings also provide important insights into the development of sustainable fisheries management for both species.

## 1 INTRODUCTION

Restricted gene flow among geographic locations can lead to genetic differentiation, therefore geographical distance is generally recognized as one of the major proxies explaining population genetic structure (Gandra et al., 2021). Because the ocean typically lacks physical barriers that disrupt gene flow, marine fish species have traditionally been considered to exhibit little genetic differentiation across locations (explained by the low‐structure–high‐gene‐flow scenario; Hemmer‐Hansen et al., 2007). Particularly, planktonic egg and larval stages of most taxonomic groups have great potential for dispersal over long distances, contributing to genetic homogenization over large spatial scales (Cowen & Sponaugle, 2009; Knutsen et al., 2022). However, recent studies have increasingly reported genetic differentiation among marine fish populations, resulting from species‐specific ecological traits and historical population demography (e.g., Alexandridis et al., 2025; Ma et al., 2018; Payet et al., 2022; Seyoum et al., 2017; Wang et al., 2024).

Population genetic differentiation can arise from various ecological and environmental factors as well as historical processes. Ecological factors include planktonic larval duration (PLD), egg type, high variation in reproductive success, selection, swimming ability, the distance between suitable habitats (habitat patchiness) and spatial scale of adult migration (D'Aloia et al., 2015; Frisk et al., 2014; Knutsen et al., 2022; Liggins et al., 2020; Riginos et al., 2011; Riginos & Liggins, 2013). Environmental factors such as ocean currents, temperature, salinity, dissolved oxygen, deep‐sea trenches, shoreline configurations and freshwater inputs can also influence patterns of genetic differentiation (Liggins et al., 2020; Riginos & Liggins, 2013). Furthermore, paleogeographic changes associated with the Pleistocene glacial–interglacial cycles have a profound impact on population demographic histories, which are closely linked to present‐day population genetic structure and diversity (Avise, 2000). For example, sea‐level oscillations have reshaped the habitats for coastal marine species through the formation and regression of land bridges. These changes can trigger population bottlenecks, founder events, temporal population isolation and subsequent secondary contacts (Avise, 2009; Maggs et al., 2008). An increasing number of studies on coastal marine species have reported that such historical population dynamics, coupled with species‐specific ecology, influence the present‐day population genetic structure, effective population size (*N*_e_) and genetic diversity across the seascape (e.g. De Jesús‐Bonilla et al., 2025; Hirase, 2022; Matsui, 2022; Ni et al., 2014).

Fish eggs are broadly classified into pelagic and demersal types based on their relative buoyancy within the water column. Some meta‐analysis studies on a wide range of marine fish taxa revealed that egg type presents a significant contribution to the level of genetic differentiation (Bradbury et al., 2008, Riginos et al., 2011, but also see Gandra et al., 2021). These studies suggested that demersal egg spawners tend to exhibit larger genetic differentiation among conspecific populations compared to pelagic egg spawners, likely due to their limited dispersal potential during early life stages. To examine specific differences in population genetic structures resulting from variations in egg types, a comparative approach focusing on closely related species at the same geographic scale is desirable because many aforementioned factors can affect population genetic structures. However, closely related species rarely exhibit divergence in egg traits, and therefore only a few comparative studies have addressed such cases. For example, Magsino and Juinio‐Meñez (2008) found that the demersal‐egg‐spawning mottled spinefoot *Siganus fuscescens* (Houttuyn 1782) exhibited greater genetic differentiation compared to the pelagic‐egg‐spawning forktail rabbitfish *Siganus argenteus* (Quoy & Gaimard 1825) along the eastern Philippine coast. Momigliano et al. (2017) investigated the population genetic structure of two groups of European flounder *Platichthys flesus* (L. 1758) that differ in egg traits in the Baltic Sea. In that study, no genetic differentiation was detected within the demersal‐egg spawning group, which was later described as a new species, the Baltic flounder *Platichthys solemdali* Momigliano, Denys, Jokinen & Merilä 2018, likely reflecting its very recent speciation event. Consequently, little is known about how differences in egg traits influence species‐specific population genetic structures, including population demographic histories.

In addition to understanding the factors driving population differentiation, knowledge of population genetic structures is required for management and conservation of natural resources (Hauser & Carvalho, 2008; Laikre et al., 2005). Particularly for fisheries target species, such information is also important for improving the accuracy of stock assessments (Cadrin et al., 2020, 2023). Therefore, identifying the management units (MUs), or stocks in fisheries, based on genetic information is crucial for preventing overexploitation of wild populations (Palsbøll et al., 2007; Schwartz et al., 2007). Failure to recognize spatial subdivisions may lead to overfishing and increase the extinction risk for local populations (Lowe & Allendorf, 2010; Spies et al., 2015; Stephenson, 1999; Ying et al., 2011). Furthermore, examining historical population dynamics associated with paleogeographic changes provides insights into the underlying evolutionary processes of present‐day population genetic structures and offers valuable information for fisheries management (Hauser & Carvalho, 2008).

This study focused on two commercially important flounders, yellow striped flounder *Pseudopleuronectes herzensteini* (Jordan & Snyder 1901) and marbled flounder *Pseudopleuronectes yokohamae* (Günther 1877). Around the Japanese Archipelago, *P. herzensteini* is distributed along the Japan Sea coast of Kyushu and the Japanese mainland, the Pacific coast from Tosa Bay and northward, and Hokkaido, while *P. yokohamae* is distributed along the East China Sea and the Japan Sea coasts from western Kyushu to central Hokkaido, and the Pacific coast from Oita to central Hokkaido (Amaoka, 2016). Both species inhabit sandy‐muddy bottoms at depths shallower than 100 m (Nakabo, 2002) and are often sympatric (Gomelyuk & Shchetkov, 1999). They exhibit contrasting early life‐history traits related to dispersal, including egg traits and the duration of planktonic stages (Table S1 and Figure S1). As is commonly observed in flatfish species, *P. herzensteini* spawns pelagic eggs near the sea bottom, where the eggs gradually float and the hatched larvae undergo ontogenetic migration to the bottom (Imura et al., 2004). Planktonic eggs and larvae are dispersed by ocean currents and coastal eddies (Nakata, 1996; Suenaga et al., 1996) within approximately 50 days (Figure S1). In contrast, *P. yokohamae* employs an early life strategy that minimizes PLD and limits dispersal by depositing adhesive demersal eggs that float only after hatching (Takatsu, 2022). This species spawns in shallower areas than *P. herzensteini* where hatched larvae are further dispersed to coastal waters through selective tidal‐stream transport and compensatory drift induced by seasonal winds (Nakagami et al., 2001; Takahashi et al., 1986) during an approximately 30‐day PLD (Figure S1). After the juvenile stage, long‐range migration is rare in both species (Amaoka, 2016; Fujii et al., 2008; Takahashi et al., 1987; Tanda, 2008), therefore these species are ideal for examining the differences in population genetic structures resulting from variations in their early life histories.

Previous population genetic studies on *P. herzensteini* reported no significant differentiation (Kim et al., 2010; Park et al., 1990). In contrast, *P. yokohamae* is likely to exhibit significant genetic differentiation even among geographically adjacent sampling locations (e.g. Gwak & Roy, 2024; Lee et al., 2012; Park et al., 1990; Sato et al., 2018; Tsukagoshi et al., 2015; Zhang et al., 2012). These studies have suggested that limited early‐life dispersal and small‐scale adult migration, together with environmental factors, may have influenced the formation of genetically distinct populations of *P. yokohamae*. However, previous studies of both species collected samples from relatively few locations and limited study areas, therefore knowledge of the range‐wide population genetic structure of these species remains limited, despite its importance for effective fisheries management. Moreover, some of these studies relied on less informative allozyme markers or partial mitochondrial DNA (mtDNA) control region, with the latter posing the risk of misinterpreting genetic diversity and population demographic histories due to homoplasy (Yamamoto et al., 2024).

A previous mtDNA‐based study suggested that the closely related species cresthead flounder *Pseudopleuronectes schrenki* (Schmidt 1904) and *P. yokohamae* hybridized primarily in northern Japan (Tsukagoshi et al., 2015). *Pseudopleuronectes schrenki* occurs in more northern waters than *P. yokohamae*, although their distribution ranges partially overlap in some areas, such as the southern part of Hokkaido (Amaoka, 2016; Nishiuchi, 1991). These two species are considered to have diverged relatively recently, and taxonomic confusion between them persisted until recent years (Kartavtsev et al., 2008; Vinnikov et al., 2006, 2018; Zhang et al., 2011). Although hybridization between them has not yet been examined using nuclear DNA markers, the presence of *P. yokohamae* individuals carrying *P*. *schrenki* mtDNA haplotypes, and vice versa, suggests the possibility of bidirectional hybridization (Tsukagoshi et al., 2015). To avoid potential bias in population genetic analyses, individuals carrying *P*. *schrenki* mtDNA haplotypes were excluded in this study.

To examine the effects of differences in early life histories including egg traits on population genetic structure, we conducted a comparative population genetic analysis of the two congeneric flounders around the Japanese Archipelago using mtDNA sequences from the NADH dehydrogenase subunit 2 (ND2) and cytochrome *b* (Cyt *b*) genes. Based on the differences in early life histories, we hypothesize that *P. yokohamae* exhibits greater intraspecific genetic differentiation compared to *P. herzensteini* due to its lower dispersal potential. In this study, a comparative population genetic analysis revealed contrasting patterns in population genetic structure and demographic history between the two species, likely reflecting differences in dispersal during early life stages.

## 2 MATERIALS AND METHODS

### 2.1 Sample collection

**FIGURE 1:**
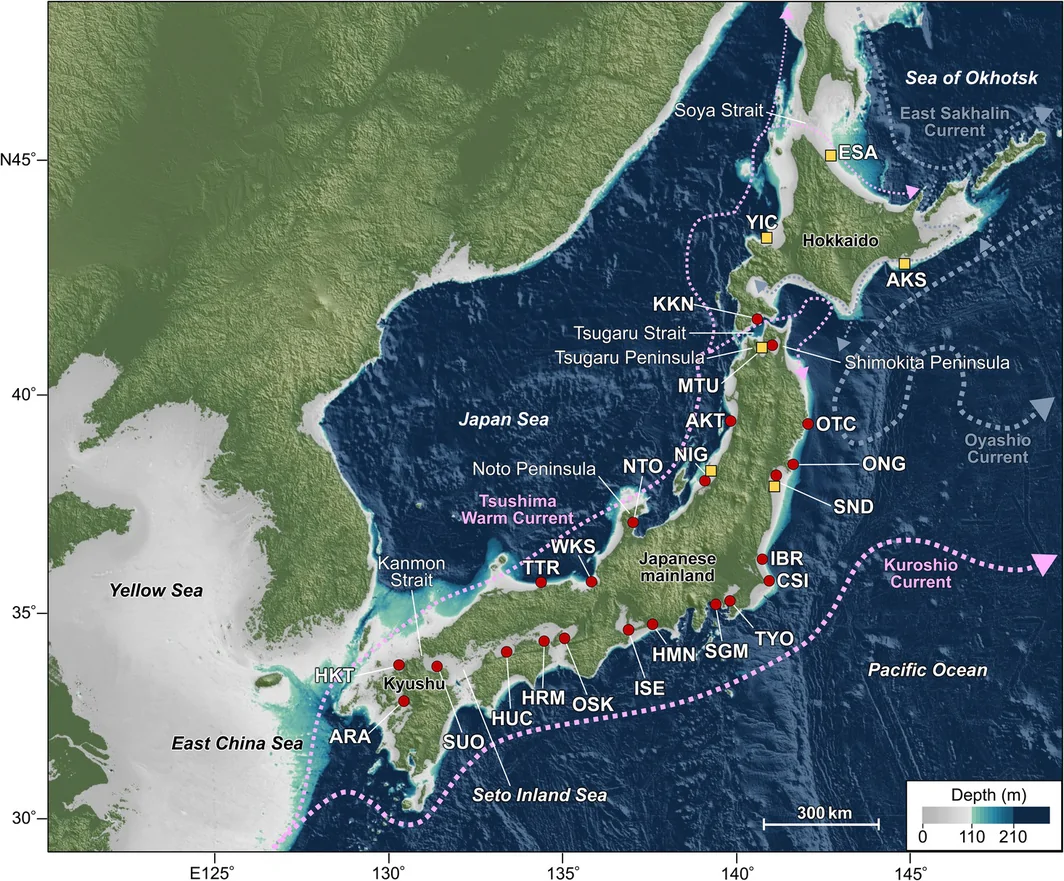
Sampling locations for *Pseudopleuronectes herzensteini* (yellow squares) and *P. yokohamae* (red circles). More information on each sampling location is provided in Table 1. Major warm and cold ocean currents around the Japanese Archipelago are illustrated with pink and blue dashed lines, respectively. Estimated terrestrial areas during the Last Glacial Maximum (depths shallower than 110 m) are represented by light grey. Areas shallower than 100 m during this period, representing potential habitats inferred from bathymetry, are shown in light blue. The map was generated using GMT6 software (Wessel et al., 2019).

A total of 279 *P. herzensteini* individuals from six locations and 1079 *P. yokohamae* individuals from 22 locations around the Japanese Archipelago were collected between 2007 and 2018 (Table 1 and Figure 1). Fin clips or muscle tissues were preserved in 99.5% ethanol and stored at −30°C.

**TABLE 1:** Sample information and genetic diversity indices for *Pseudopleuronectes herzensteini* and *P. yokohamae*. Groupings in *P. yokohamae* were inferred using spatial analysis of molecular variance (SAMOVA).

Species	Locality (abbreviation)	Sampling year	Sampling method	*n*	nCF	nHp	nPs	*h*	*π* (× 100)
*P. herzensteini*	Niigata (NIG)	2016	Fisheries	46	–	37	73	0.990	0.605
	Mutsu Bay (MTU)	2016	Fisheries	47	–	41	85	0.994	0.652
	Yoichi (YIC)	2017	Fisheries	48	–	44	85	0.996	0.619
	Esashi (ESA)	2016	Fisheries	44	–	35	74	0.985	0.591
	Akkeshi (AKS)	2016	Fisheries	47	–	39	78	0.993	0.610
	Sendai Bay (SND)	2016	Fisheries	47	–	37	72	0.990	0.628
			Total	279	–	160	182	0.992	0.617
*P. yokohamae*	Kyushu group								
	Ariake Sea (ARA)	2016–17	Angling, Fisheries	51	0	21	34	0.802	0.240
	Hakata Bay (HKT)	2016–17	Angling	23	0	7	12	0.806	0.210
			Subtotal	74	0	24	38	0.820	0.235
	Japan Sea group								
	Tottori (TTR)	2016–17	Angling	18	0	10	13	0.915	0.211
	Wakasa Bay (WKS)	2016	Fisheries	50	0	21	38	0.900	0.274
	Noto Island (NTO)	2016–17	Angling, Fisheries	15	0	10	18	0.924	0.254
	Niigata (NIG)	2016	Fisheries	45	1	18	32	0.902	0.229
	Akita (AKT)	2018	Fisheries	30	0	17	36	0.926	0.296
	Mutsu Bay (MTU)	2012–13	Fisheries	47	3	19	36	0.926	0.250
	Kikonai Bay (KKN)	2013–14, 2016	Fisheries	83	16	29	41	0.897	0.253
			Subtotal	288	20	69	81	0.925	0.260
	Tohoku Pacific group								
	Otsuchi Bay (OTC)	2012–13	Fisheries	120	1	34	51	0.943	0.325
	Onagawa Bay (ONG)	2016	Angling	106	0	48	59	0.966	0.338
	Sendai Bay (SND)	2016	Fisheries	66	1	34	51	0.951	0.339
	Ibaraki (IBR)	2013	Fisheries	44	1	30	45	0.968	0.355
	Choshi (CSI)	2007	Fisheries	41	0	24	39	0.963	0.309
			Subtotal	377	3	92	95	0.964	0.335
	Kanto group								
	Tokyo Bay (TYO)	2008	Fisheries	48	0	16	29	0.888	0.274
	Sagami Bay (SGM)	2008	Fisheries	24	0	13	21	0.895	0.200
			Subtotal	72	0	21	33	0.895	0.250
	Tokai group								
	Lake Hamana (HMN)	2009	Fisheries	44	0	4	4	0.215	0.032
	Ise Bay (ISE)	2008	Fisheries	32	0	11	10	0.534	0.050
			Subtotal	76	0	14	12	0.357	0.040
	Seto Inland Sea group								
	Osaka Bay (OSK)	2016	Fisheries	45	0	20	34	0.924	0.382
	Harima‐nada Sea (HRM)	2017	Fisheries	47	0	22	40	0.928	0.394
	Hiuchi‐nada Sea (HUC)	2017	Fisheries	48	0	20	36	0.928	0.398
	Suo‐nada Sea (SUO)	2016–17	Angling, Fisheries	52	0	26	40	0.956	0.393
			Subtotal	192	0	51	65	0.932	0.389
			Total	1079	23	223	180	0.969	0.346

Abbreviations: *h*, haplotype diversity; *n*, sample size; nCF, number of individuals carrying *P*. *schrenki* mitochondrial DNA haplotypes; nHp, number of haplotypes; nPs, number of polymorphic sites; *π*, nucleotide diversity.

### 2.2 Ethics statement

The samples were treated according to the guidelines for animal ethics in sampling from wild fauna (https://www.fra.go.jp/shigen/publication/2024-02/fri_animal_ethics_guidelines.pdf).

### 2.3 mtDNA sequencing

Total genomic DNA was extracted using the QuickGene DNA tissue kit S and Mini80 (Kurabo) or Wizard® SV 96 Genomic DNA Purification System (Promega) following the manufacturer's instructions. The target fragments of the mtDNA markers, the first half of ND2 (750 bp) and Cyt *b* (750 bp) were sequenced. In addition, the first half of mtDNA control region data was obtained for *P. yokohamae* samples to exclude individuals carrying haplotypes of closely related *P. schrenki* (see Section 2.4). Details of the sequencing methods, including PCR conditions and primer sets used, are provided in Yamamoto et al. (2024). The sequences were manually edited using FinchTV 1.4.0 (Geospiza; http://www.geospiza.com) and aligned using ClustalW implemented in MEGA7 (Kumar et al., 2016). All sequences obtained in this study were deposited in the GenBank/EMBL/DDBJ databases (Accession numbers LC882785–LC883537).

### 2.4 Detection of *P. schrenki* haplotypes

A previous study suggested that closely related *P. yokohamae* and *P*. *schrenki* hybridized primarily in northern Japan using mtDNA control region data (Tsukagoshi et al., 2015). To avoid potential bias in population genetic analyses in this study, individuals of *P. yokohamae* that were placed within the *P*. *schrenki* clade in the neighbour‐joining (NJ) tree (Saitou & Nei, 1987) were excluded based on mtDNA control region data in light of that study. The exclusion procedure is described in detail in Yamamoto et al. (2024).

### 2.5 Analyses of genetic diversity and population genetic structure

The sequences of ND2 and Cyt *b* regions were concatenated for further population genetic analyses of *P. herzensteini* and *P. yokohamae*.

The haplotype diversity (*h*; Nei, 1987) and nucleotide diversity (*π*; Nei & Li, 1979) were computed using Arlequin ver. 3.5.2. (Excoffier & Lischer, 2010). To assess genetic differentiation among the sampling locations, an analysis of molecular variance (AMOVA; Excoffier et al., 1992) was performed using Arlequin with 10,000 permutations. Pairwise *F*_ST_ values (Weir & Cockerham, 1984) were also computed, and the significance level (*α* = 0.05) for multiple comparisons was adjusted using the sequential Bonferroni correction (Rice, 1986).

If significant genetic differentiation was detected in AMOVA, population genetic structure was further examined using isolation‐by‐distance (IBD; Wright, 1943) analyses, non‐metric multidimensional scaling (NMDS), BARRIER (Manni et al., 2004), spatial analysis of molecular variance (SAMOVA; Dupanloup et al., 2002) and hierarchical AMOVA. First, the Mantel test (Mantel, 1967) was performed using the R ade4 package (Dray & Dufour, 2007) with 10,000 permutations to test for IBD by correlating the genetic distance [*F*_ST_/(1 − *F*_ST_)] with geographical distance (coastal distance) between locations. Geographical distances between sampling locations were measured along the coastline using Google Maps (https://maps.google.com/) at a scale of 5 km. To account for the potential type II error associated with the Mantel test, the relationship between genetic and geographical distances was also examined using simple linear regression implemented in R. Second, NMDS was performed using R, based on genetic distances (*D*_A_; Nei, 1987) among the sampling locations generated in Arlequin. Model suitability was tested using the stress value calculated with the isoMDS() function of the MASS package in R. Third, the pattern of genetic differentiation was further assessed using BARRIER and SAMOVA, incorporating the geographic information of each sampling location. For the BARRIER analysis, the Monmonier algorithm was implemented in BARRIER ver. 2.2 to identify genetic barriers to gene flow between geographically adjacent sampling locations. Genetic distances (*D*_A_) were used as the input matrix, and genetic barriers were subsequently identified until one was no longer supported by significant pairwise *F*_ST_ values (*α* = 0.05). SAMOVA provides population subdivisions (*K*, *K* ≥ 2) that are geographically homogeneous and maximally differentiated from each other, using a simulated annealing algorithm. The best partition was determined by the highest total genetic variance index (*F*_CT_) score between *K* genetic groups using SAMOVA 2.0. Further population partitioning was not applied when the group consisted of a single sampling location or when the *F*_CT_ value was not significant. If any of the output groups comprised more than three sampling locations, SAMOVA was repeatedly performed on the group until no significant underlying structure was detected. Finally, the hierarchical distribution of genetic variation among and within the identified groups was examined using AMOVA.

To evaluate the genealogical relationships of haplotypes, median‐joining haplotype networks (Bandelt et al., 1999) were constructed using NETWORK 5.0.1.1 (http://www.fluxus-engineering.com/sharenet.htm). The level of genetic mixture of individuals was estimated using the Bayesian analysis of population structure (BAPS) 6.0 program (Corander et al., 2008; Corander & Tang, 2007). This analysis identifies the optimal number of clusters (*k*) based on the maximum posterior probability, which enables a comparison of the cluster compositions among sampling locations. The analysis was performed using *population mixture analysis* and *clustering with linked loci* options, with *k* ranging from 1 to 6 for *P. herzensteini* and 1 to 22 for *P. yokohamae*, corresponding to the number of sampling locations. Differences in cluster composition ratios were evaluated using the chi‐square or Fisher's exact test in R. To identify the phylogenetic relationships among the *k* clusters, a non‐rooted NJ tree was constructed from the BAPS output file based on Kullback–Leibler divergence among clusters, using PHYLIP ver. 3.695 (Felsenstein, 2005) and MEGA7.

### 2.6 Analyses of population demographic history

Historical demographic analyses were performed using all individuals of *P. herzensteini* pooled across locations, whereas analyses for *P. yokohamae* were conducted separately for each of the six genetic groups identified in the population genetic structure analyses (see Section 3.3). To assess departures from neutrality and historical demographic patterns, neutrality tests were performed. To account for differences in sensitivity and robustness to sample size among tests, three statistics, Tajima's *D* (Tajima, 1989), Fu's *F*_S_ (Fu, 1997), and Ramos‐Onsins and Rozas's *R*_2_ (Ramos‐Onsins & Rozas, 2002) were calculated. Tajima's *D* and Fu's *F*_S_ were calculated in Arlequin, whereas *R*_2_ was computed using DnaSP ver. 6.12.03 (Rozas et al., 2017), with statistical significance evaluated using 1000 coalescent simulations. Deviations from neutrality may result from demographic processes (i.e. population expansion) or selection.

To infer historical demographic events, median‐joining haplotype networks were constructed using NETWORK and private haplotypes (endemic to a population) were identified. Mismatch distribution analysis (Rogers & Harpending, 1992) was performed in Arlequin to further evaluate historical demographic patterns. The observed distribution of pairwise differences was compared with that expected under a simulated sudden expansion model using 1000 bootstrap replications. Goodness of fit was evaluated using the sum of squared deviations (SSD) and Harpending's raggedness index (Harpending, 1994; Harpending et al., 1993). Demographic scenarios such as population expansions, bottlenecks or selective sweeps can be inferred from haplotype networks and observed mismatch distributions (Avise, 2000; Harpending et al., 1998; Rogers & Harpending, 1992).

A Bayesian skyline plot (BSP; Drummond et al., 2005) was performed to explore temporal trends in population demographic history. Skyline‐plot methods are based on the coalescent theory to reconstruct fluctuations in *N*_e_ (female effective population size *N*_ef_ for mtDNA data) over time with information contained in haplotype genealogies of DNA sequences (Ho & Shapiro, 2011). Sequences were partitioned by region, with priors and parameters assigned individually. This analysis was performed in BEAST v2.7.6 (Bouckaert et al., 2014) under a strict clock model and a coalescent Bayesian skyline model, with nucleotide substitution models estimated by MEGA7 (Table S2). A general mutation rate of 2.0% per million years (Myr) for bony fish, or 0.01 substitutions per site per lineage per Myr (Bermingham et al., 1997), was applied for both ND2 and Cyt *b* regions, as the specific rates for the studied species were unavailable. The clock rate prior was set to a log‐normal distribution with *M* = 1.0 × 10^−8^ and *S* = 0.25 for both regions. A Markov chain Monte Carlo analysis was performed using Tracer v1.7.2 (Rambaut et al., 2018) with sufficient generations to ensure that all effective sample size values exceeded 200 (Table S2), sampling every 1000 generations and discarding the first 10% as burn‐in. BSPs were then generated using Tracer. A generation time of 3 years was used for both species to calculate *N*_ef_, based on the age at which more than 50% of adults reach sexual maturity (Takahashi et al., 1983).

## 3 RESULTS

### 3.1 Detection of *P. schrenki* haplotypes

Using the control region sequences (332 bp) from 1079 *P. yokohamae* individuals, 62 *P. schrenki* haplotypes obtained by Tsukagoshi et al. (2015), and five *P. herzensteini* individuals as an outgroup, 345 haplotypes were identified. A total of 23 *P. yokohamae* individuals from NIG, MTU, KKN, OTC, SND and IBR sampling locations were classified as *P. schrenki* haplotypes (Table 1 and Figure S2), thus the remaining 1056 individuals were used for subsequent analyses.

### 3.2 Population genetic structure of *P. herzensteini*

**FIGURE 2:**
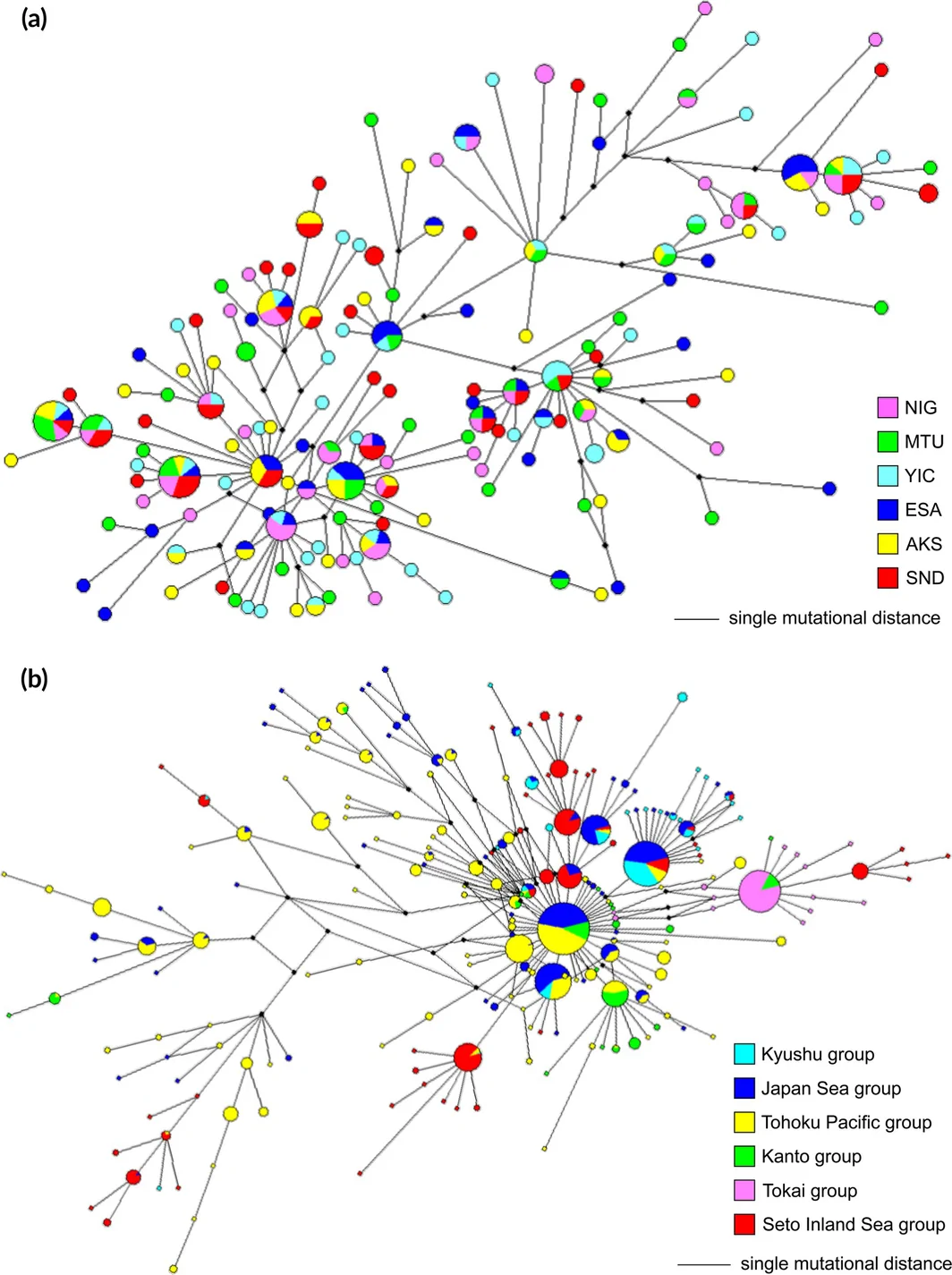
Median‐joining haplotype networks based on mitochondrial DNA sequences showing (a) 160 haplotypes for *Pseudopleuronectes herzensteini* and (b) 223 haplotypes for *P. yokohamae*. Haplotypes are coloured by sampling location in *P*. *herzensteini* and by genetic group in *P. yokohamae* (see Table 1 for sampling locations and group assignments). Branch lengths are proportional to the number of substitutions. Node sizes are also proportional to the number of individuals (*n*) observed for each haplotype (smallest circle, *n* = 1; largest circles, *n* = 10 in *P. herzensteini* and *n* = 109 in *P. yokohamae*, respectively). Median vectors are displayed as solid squares.

**FIGURE 3:**
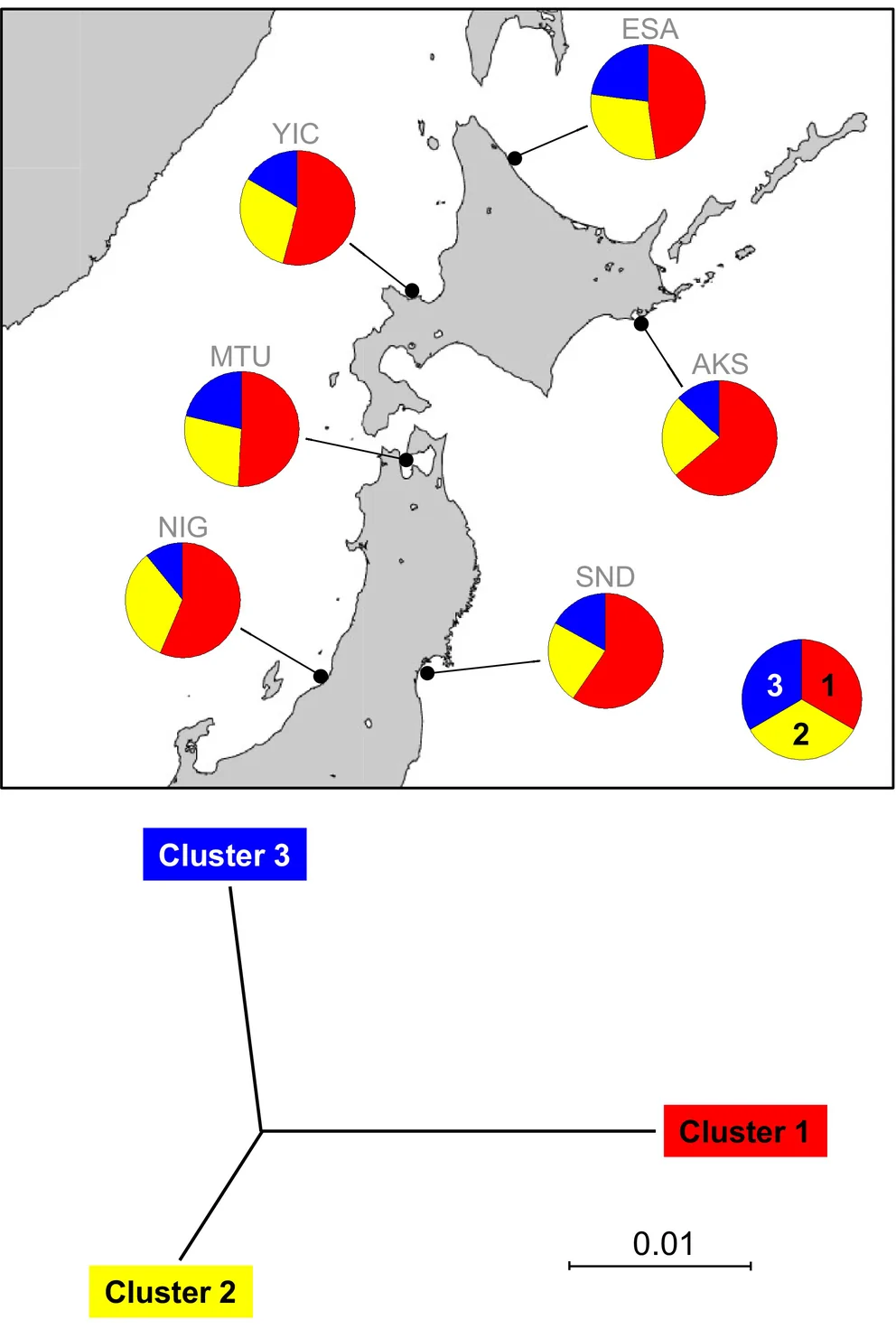
Population genetic structure of *Pseudopleuronectes herzensteini* inferred from individual‐based Bayesian analysis of population structure. Pie charts represent the mixture of individuals for each location, assigned to the optimal number of three genetic clusters. Abbreviations of sampling locations are shown in grey (see Table 1). The genetic relationship of the BAPS clusters is shown below with a non‐rooted neighbour‐joining tree based on the estimated Kullback–Leibler divergence among them.

The genetic diversity was high in all sampling locations of *P. herzensteini* with *h* ≥ 0.98 and *π* × 100 ≥ 0.59, respectively (Table 1). The AMOVA failed to detect significant genetic differentiation among the six sampling locations (global *F*_ST_ = −0.002, *p* = 0.70; Table 2). All pairwise *F*_ST_ values were non‐significant, ranging from −0.009 to 0.008 (Figure S3a). The haplotype network exhibited a diffuse pattern, with individuals from each location widely distributed across the network (Figure 2a). Genetic mixture analysis using BAPS identified the optimal *k* = 3 (log‐likelihood = −4734.7957) without highly divergent BAPS clusters (Figure 3). There was no significant difference in BAPS cluster composition among sampling locations (chi‐square test: *χ* ^2^ = 5.41, *df* = 10, *p* = 0.86).

**TABLE 2:** Analysis of molecular variance (AMOVA) of mitochondrial DNA haplotypes in *Pseudopleuronectes herzensteini* and *P. yokohamae*. Hierarchical AMOVA for *P. yokohamae* is presented based on the genetic groupings (See Table 1).

Species	Comparison	Source of variation	*df*	Sum of squares	Variance components	Percentage of variation	*F*‐statistics
*P. herzensteini*	All locations	Among locations	5	20.51	−0.011	−0.25	*F*_ST_ = −0.002
		Within locations	273	1265.08	4.634	100.25	
*P. yokohamae*	All locations	Among locations	21	491.71	0.448	17.08	*F*_ST_ = 0.171***
		Within locations	1034	2248.89	2.175	82.92	
	Six genetic groups	Among groups	5	443.00	0.531	19.49	*F*_CT_ = 0.195***
		Among locations within groups	16	48.71	0.019	0.68	*F*_SC_ = 0.008**
		Within locations	1034	2248.89	2.175	79.83	*F*_ST_ = 0.202***
	Kyushu group	Among locations	1	3.80	0.065	3.63	*F*_ST_ = 0.036
		Within locations	72	124.61	1.731	96.37	
	Japan Sea group	Among locations	6	21.75	0.046	2.38	*F*_ST_ = 0.024***
		Within locations	261	497.94	1.908	97.62	
	Tohoku Pacific group	Among locations	4	16.33	0.022	0.88	*F*_ST_ = 0.009*
		Within locations	369	920.93	2.496	99.12	
	Kanto group	Among locations	1	1.90	0.001	0.05	*F*_ST_ = 0.000
		Within locations	70	131.25	1.875	99.95	
	Tokai group	Among locations	1	0.46	0.004	1.45	*F*_ST_ = 0.014
		Within locations	74	21.80	0.295	98.55	
	Seto Inland Sea group	Among locations	3	4.48	−0.030	−1.04	*F*_ST_ = −0.010
		Within locations	188	552.37	2.938	101.04	

Abbreviations: *F*_CT_, proportion of total genetic variance among groups; *F*_SC_, proportion of genetic variance among sampling locations within groups; *F*_ST_, proportion of total genetic variance among sampling locations.

* *p* < 0.05.

** *p* < 0.01.

*** *p* < 0.001 for *F*‐statistics.

### 3.3 Population genetic structure of *P. yokohamae*

Genetic diversity was high at most sampling locations for *P. yokohamae* with *h* ≥ 0.9 and *π* × 100 ≥ 0.2 (Table 1); however, HMN (*h* = 0.215, *π* × 100 = 0.032) and ISE (*h* = 0.534, *π* × 100 = 0.050) showed lower genetic diversity compared to the other locations. The global AMOVA revealed significant overall genetic differentiation (global *F*_ST_ = 0.171, *p* < 0.001), with 17.1% of variation among the 22 locations and 82.9% within locations (Table 2). Among the 231 pairs of sampling locations, 156 (67.5%) showed significant pairwise *F*_ST_ values following sequential Bonferroni correction (initial *α* = 0.00022; Figure S3b). Pairwise *F*_ST_ values involving HMN or ISE were remarkably high compared to other locations, with a maximum of 0.760 between TTR and HMN.

**FIGURE 4:**
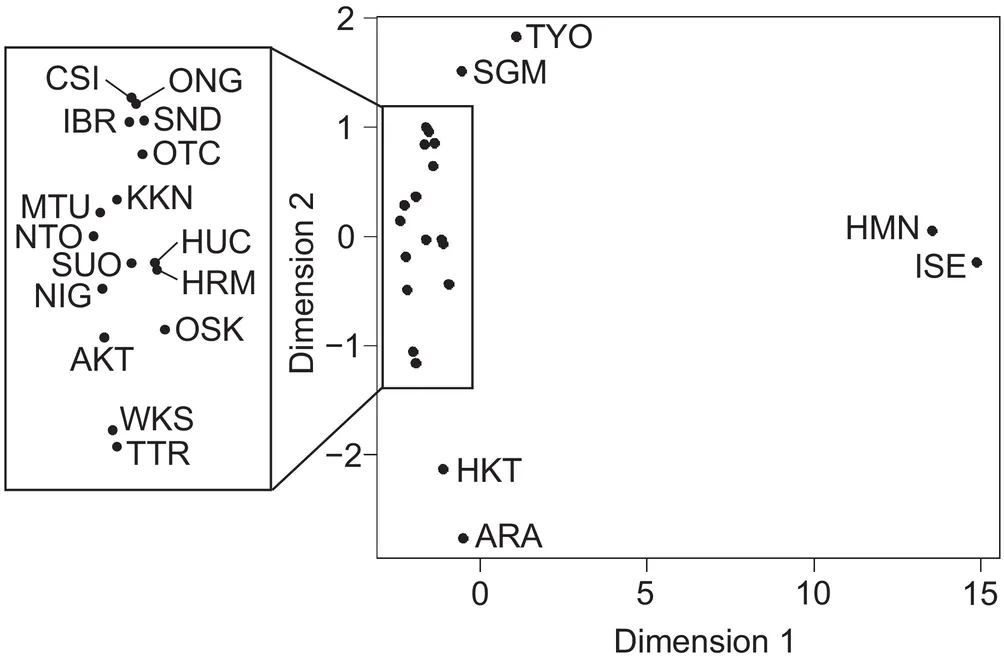
Non‐metric multidimensional scaling plot based on *D*_A_ genetic distances among the 22 sampling locations of *Pseudopleuronectes yokohamae*. See Table 1 for abbreviations of sampling locations.

No significant IBD pattern was detected across sampling locations by either the Mantel test (*r* = 0.114, *p* = 0.064; Figure S4a) or linear regression (*R* ^2^ = 0.013, *p* = 0.084). The NMDS plot (stress = 0.0296) identified three geographically distinct groups apart from the predominant group of plots (Figure 4): ARA and HKT (Kyushu group), TYO and SGM (Kanto group), and HMN and ISE (Tokai group). The scaled‐up plots also showed that geographically closer sampling locations were grouped together.

**FIGURE 5:**
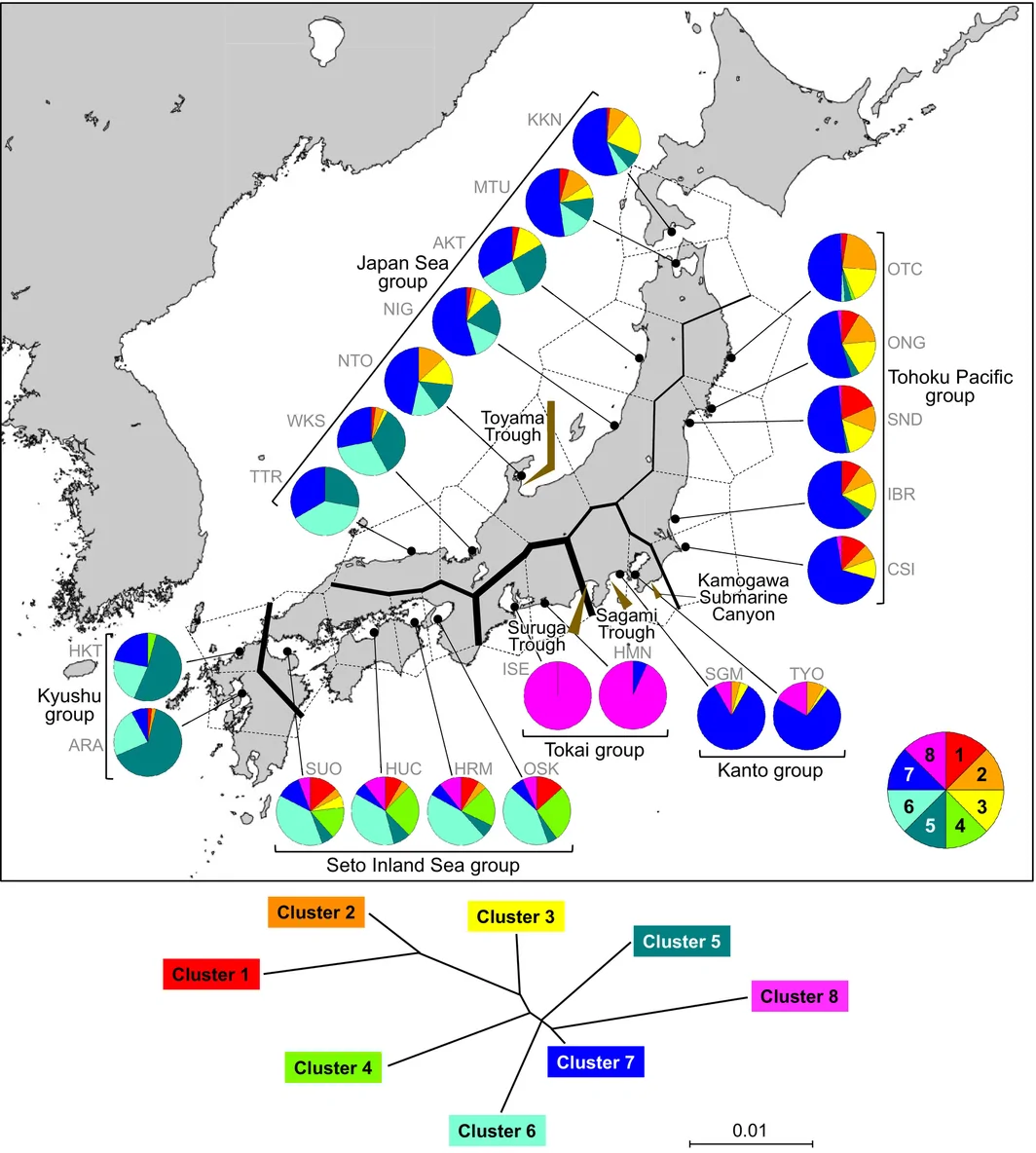
Population genetic structure of *Pseudopleuronectes yokohamae* inferred from BARRIER, spatial analysis of molecular variance (SAMOVA) and Bayesian analysis of population structure (BAPS). Genetic barriers to gene flow between geographically adjacent sampling locations are identified using BARRIER, represented by solid lines over the Voronoi polygons formed by dashed lines. Line thickness corresponds to the level of genetic differentiation. Pie charts represent the mixture of individuals for each location, assigned to the optimal number of eight genetic clusters using an individual‐based analysis with BAPS. Abbreviations of sampling locations are shown in grey (see Table 1). The genetic relationship of the BAPS clusters is shown below with a non‐rooted neighbour‐joining tree based on the estimated Kullback–Leibler divergence among them. The displayed groupings were inferred by SAMOVA.

Possible barriers to gene flow identified using BARRIER were generally consistent with patterns observed in the NMDS plot (Figure 5). Primary genetic discontinuities were identified surrounding the Tokai group. The second largest barrier was detected around the Kyushu group. Barriers to gene flow were identified around Seto Inland Sea (OSK, HRM, HUC and SUO) and Kanto groups, with additional weaker barriers detected between MTU and OTC.

SAMOVA identified seven genetic groups among sampling locations. Initially, the 22 sampling locations were split into the Tokai group and remaining 20 locations (*K* = 2, *F*_CT_ = 0.408, *p* = 0.003). SAMOVA was then performed again on the 20 sampling locations, and the Kyushu group was separated from the remaining locations (*K* = 2, *F*_CT_ = 0.106, *p* = 0.006). The 18 remaining sampling locations were further divided into four groups (*K* = 4, *F*_CT_ = 0.094, *p* < 0.001): Japan Sea group 1 (TTR, WKS and AKT), the Kanto group, the Seto Inland Sea group and nine remaining locations. Finally, the remaining locations were separated into Japan Sea group 2 (NTO, NIG, MTU and KKN) and Tohoku Pacific group (OTC, ONG, SND, IBR and CSI), with *K* = 2 (*F*_CT_ = 0.030, *p* = 0.011). No subgroups were detected within the Seto Inland Sea group, Japan Sea group 2 or Tohoku Pacific group.

SAMOVA clustering split the sampling locations along the Japan Sea coast into geographically inconsistent groups, the Japan Sea groups 1 and 2, thus we further examined the population genetic structure of these groups. AMOVA showed that 2.38% of the variation was attributed to differences among the seven Japan Sea sampling locations (*p* < 0.001); however, *F*_ST_ was not exceptionally high compared to other genetic groups (Table 2). Only one pair of sampling locations (WKS and KKN) exhibited significant pairwise *F*_ST_ values following sequential Bonferroni correction (Figure S3b). No genetic barrier was detected around the Japan Sea coast based on the BARRIER analysis (Figure 5), and a significant IBD pattern was identified across the seven Japan Sea locations based on both the Mantel test (*r* = 0.577, *p* = 0.020; Figure S4b) and simple linear regression (*R* ^2^ = 0.333, *p* = 0.006). The association between genetic and geographical distances became stronger when the Tohoku Pacific group samples were included (Mantel test: *r* = 0.760, *p* < 0.001; linear regression: *R* ^2^ = 0.577, *p* < 0.001). Collectively, the seven Japan Sea locations—TTR, WKS, NTO, NIG, AKT, MTU and KKN—were considered as a single genetic group (hereafter, Japan Sea group).

Hierarchical AMOVA for the six genetic groups (Kyushu, Japan Sea, Tohoku Pacific, Kanto, Tokai and Seto Inland Sea groups) partitioned 19.5% of the total variation among the groups (*F*_CT_ = 0.195, *p* < 0.001), 0.68% among sampling locations within groups (*F*_SC_ = 0.008, *p* = 0.003) and 79.8% within sampling locations (*F*_ST_ = 0.202, *p* < 0.001; Table 2).

The haplotype network displayed a diffuse shape with star‐like patterns on its branches (Figure 2b). The most predominant haplotype consisted of 109 individuals from the Japan Sea, Tohoku Pacific and Kanto groups. Most of the major haplotypes were composed of individuals from a few groups. Each genetic group had a substantial number of private haplotypes, with haplotypes in the Tokai and Seto Inland Sea groups likely being unique to those groups.

The genetic mixture analysis with BAPS identified the optimal *k* = 8 for the 22 sampling locations (log‐likelihood = −8918.3478) without highly divergent clusters (Figure 5). BAPS cluster composition differed significantly among the sampling locations (Fisher's exact test: *p* < 0.001), showing clear differences in the cluster composition among groups and high similarity within each group. However, similar cluster compositions were observed at some sampling locations across genetic group boundaries (e.g. between HKT and TTR, and MTU and OTC). In the Tokai group, most haplotypes were categorized as the BAPS cluster 8, except for three individuals in the cluster 7 at HMN. The BAPS cluster 4 was nearly exclusive to the Seto Inland Sea group, except for its occurrence at HKT (*n* = 1) and OTC (*n* = 2) outside this group.

### 3.4 Population demographic history of *P. herzensteini*

**FIGURE 6:**
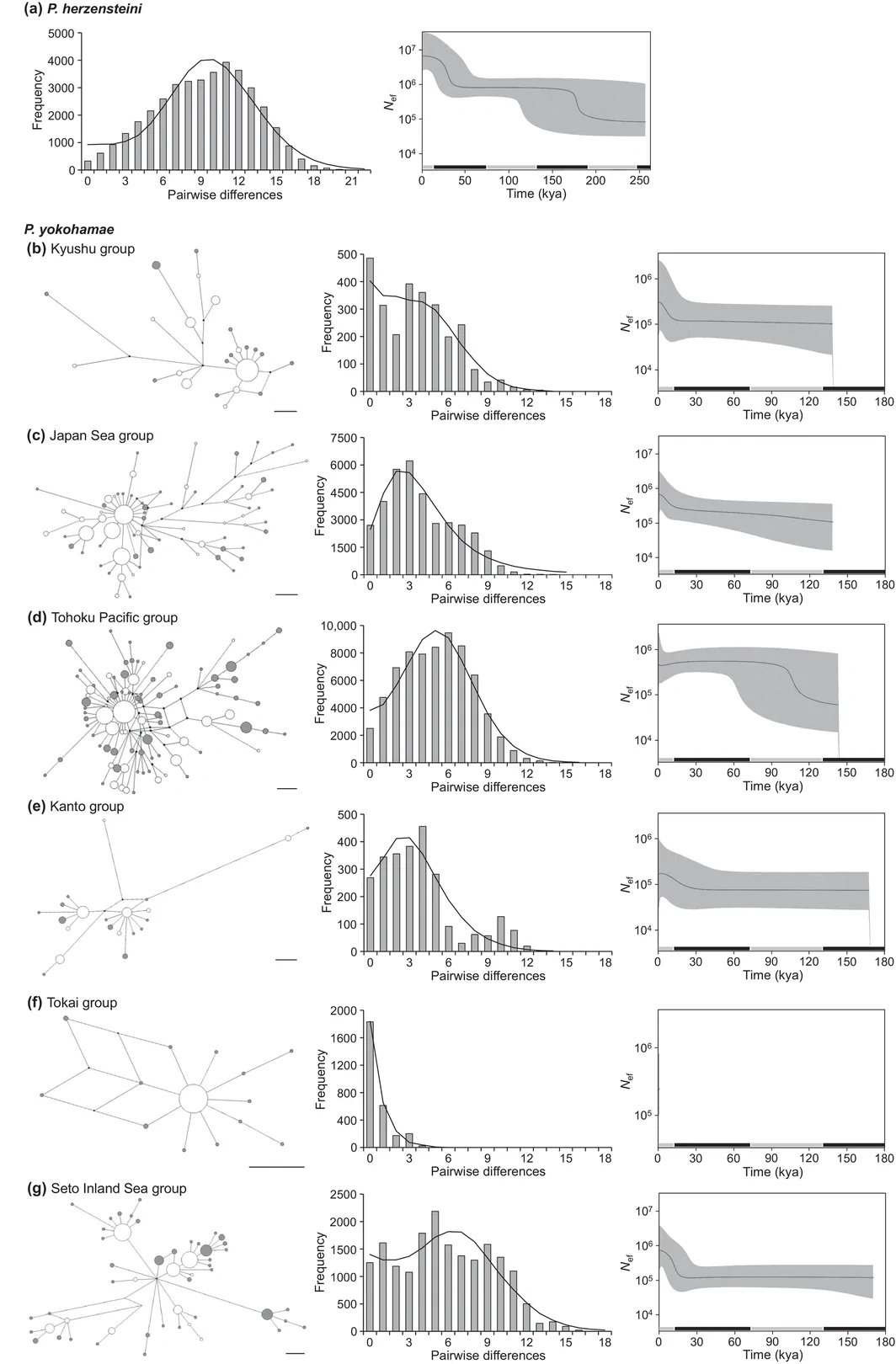
Population demographic histories of (a) *Pseudopleuronectes herzensteini* and (b–g) each genetic group of *P. yokohamae* inferred from the median‐joining haplotype networks (left), mismatch distributions (center) and Bayesian skyline plots (BSPs) (right). Note that the haplotype network of *P. herzensteini* is provided in Figure 2a. In each haplotype network, scale bar represents single mutational distance. Node sizes are proportional to the number of individuals (*n*) observed for each haplotype (smallest circle, *n* = 1; largest circles: (b) Kyushu group, *n* = 30; (c) Japan Sea group, *n* = 47; (d) Tohoku Pacific group, *n* = 50; (e) Kanto group, *n* = 17; (f) Tokai group, *n* = 61; (g) Seto Inland Sea group, *n* = 31). Median vectors are shown in solid squares. In mismatch distribution histogram, bars and lines represent observed distribution and expected distribution under the sudden expansion model, respectively. BSPs show the median estimates for female effective population size (*N*_ef_) over time (solid lines) with 95% central posterior density intervals (grey area). The x‐axis shows time before present in thousand years (kya). The black and grey bars above the x‐axis represent glacial and interglacial periods (Machida et al., 2003), respectively.

As no significant genetic differentiation was detected among sampling locations of *P. herzensteini*, individuals were pooled across locations for historical demographic analyses. All three neutrality tests (Tajima's *D*, Fu's *F*_S_, and Ramos‐Onsins and Rozas's *R*_2_) showed significant deviations from neutrality (Table S3). The haplotype network exhibited a diffuse shape with loop structures, with some terminal haplotypes showing star‐like patterns (Figure 2a). In the mismatch analysis, the sudden expansion model was supported by non‐significant SSD and raggedness index (Table S3). The mismatch distribution showed a unimodal pattern (Figure 6a). The BSP showed that *P. herzensteini* maintained a large *N*_ef_ over time, with two episodes of population expansion occurring around 180 and 30 thousand years ago (kya) (Figure 6a).

### 3.5 Population demographic history of *P. yokohamae*

Population demographic histories were inferred for each of the six genetic groups of *P. yokohamae*. Significant departures from neutrality were detected across all genetic groups in all three tests (Table S3). The haplotype networks, mismatch distributions and BSPs exhibited distinct patterns among the groups (Figure 6b–g). In the mismatch analysis, the sudden expansion model was supported by non‐significant SSDs and raggedness indices in all groups (Table S3).

Haplotype networks in the Kyushu and Seto Inland Sea groups exhibited a bottleneck shape lacking an ancestral haplotype, with additional star‐like patterns observed on some branches (Figure 6b,g). The Seto Inland Sea group predominantly consisted of unique haplotypes connected by relatively deep linkages. The Kyushu group showed a bimodal mismatch distribution, whereas the Seto Inland Sea group exhibited a multimodal mismatch distribution. The BSPs showed that both groups have maintained a constant *N*_ef_ over time, with a sharp recent increase occurring around 15 kya.

Both the Japan Sea and Tohoku Pacific groups exhibited a diffuse pattern in their haplotype networks, with a limited number of missing haplotypes and some star‐like phylogenies on their branches (Figure 6c,d). Their mismatch distributions showed a unimodal distribution. The BSPs indicated a population expansion beginning approximately 10 kya in the Japan Sea group and approximately 110 kya in the Tohoku Pacific group, with the latter maintaining a large *N*_ef_ thereafter.

The Kanto group exhibited a star‐like haplotype network, although it contained divergent haplotypes (Figure 6e). The mismatch distribution showed a unimodal pattern with a small peak at 10 pairwise differences, presumably due to divergent haplotypes. The BSP exhibited a gradual increase in recent *N*_ef_.

The Tokai group exhibited a simple star‐like haplotype network, mostly composed of unique haplotypes (Figures 2b and 6e). The mismatch analysis showed an L‐shaped mismatch distribution. The BSP indicated a population history of only approximately 900 years.

## 4 DISCUSSION

This study revealed contrasting population genetic structures between the two congeneric flounders along the Japanese Archipelago based on mtDNA data. *Pseudopleuronectes herzensteini* showed no significant genetic differentiation among the sampling locations, indicating high gene flow across the sampling area. In contrast, substantial genetic differentiation was detected among the sampling locations of *P. yokohamae*, resulting in the six genetic groups. Furthermore, each genetic group exhibited distinctive historical demographic patterns. Although the overall level of intraspecific genetic differentiation was broadly consistent with previous studies conducted at relatively restricted geographic scales, this study revealed clear contrasts in population genetic structure at a comparable geographic scale.

### 4.1 Effects of early life‐history traits on genetic differentiation

The observed difference in the level of intraspecific genetic differentiation is consistent with our hypothesis, indicating that early life‐history traits related to dispersal strongly influenced their population genetic structures. Generally, planktonic egg or larval stages have a major influence on gene flow in marine species due to their remarkable potential for passive dispersal over long distances (Cowen & Sponaugle, 2009). Post‐larval factors, such as adult migration, can also potentially affect the level of population connectivity (Yanagimoto et al., 2012). Because the studied species rarely migrate over long distances beyond the juvenile stage, these factors can be considered negligible, allowing us to focus on the effects of the differences in early life histories on population genetic structures. *Pseudopleuronectes herzensteini* spawns pelagic eggs off the coast and exhibited a longer PLD compared to *P. yokohamae*, which results in a greater dispersal potential facilitated by coastal currents (Figure S1). In contrast, *P. yokohamae* does not undergo passive egg transport, as its adhesive demersal eggs attach to substrates on the seabed. Compared to *P. herzensteini*, *P. yokohamae* produces slightly smaller eggs that hatch into relatively large larvae, which settle at smaller sizes, resulting in an overall shorter PLD (Table S1 and Figure S1a). Furthermore, *P. yokohamae* spawns in shallower areas compared to *P. herzensteini*, enabling its larvae to drift to further shallower areas through tidal or region‐specific currents, potentially reducing the risk of transport into unsuitable offshore environments (Takatsu, 2022). Collectively, these findings suggest that the differences in egg traits and planktonic dispersal during early life stages result in the contrasting population genetic structures.

Previous population genetic studies using mtDNA markers have suggested that most flatfish species exhibit extensive gene flow around the Japanese Archipelago (e.g. Kojima et al., 2014; Misawa et al., 2023; Shigenobu et al., 2007; Xiao et al., 2010). Only five demersal‐egg‐spawning flatfish species inhabit brackish or coastal waters around the Japanese Archipelago (Minami, 2008; Rijnsdorp et al., 2015): *P. yokohamae*, *P*. *schrenki*, black flounder *Pseudopleuronectes obscurus* (Herzenstein 1890), dusky sole *Lepidopsetta mochigarei* Snyder 1911 and northern rock sole *Lepidopsetta polyxystra* Orr & Matarese 2000. Among the demersal‐egg spawners, population genetic structure has been investigated only in *P*. *schrenki* apart from *P. yokohamae*. *Pseudopleuronectes schrenki* indeed showed relatively large genetic differentiation among sampling locations (Tsukagoshi et al., 2015) compared to other flatfish species that spawn pelagic eggs. The production of demersal eggs is considered an adaptation that prevents eggs and larvae from settling in offshore regions (Rijnsdorp et al., 2015). In light of the results of this study and previous findings discussed herein, life‐history strategies in demersal‐egg spawners that limit offshore dispersal of eggs and larvae may be associated with substantial genetic differentiation among populations.

We infer that differences in their early life histories primarily result in the contrasting population genetic structures; however, this interpretation should be made with caution because other factors may also be involved. First, apart from ecological factors, population genetic structure can be influenced by environmental factors, such as ocean currents and temperature (Liggins et al., 2020; Riginos et al., 2011; Riginos & Liggins, 2013). Genome‐wide environmental association studies in flatfish species have reported strong relationships between population genetic structures and oceanographic factors, such as salinity and dissolved oxygen (do Prado et al., 2018; Kess et al., 2021). Second, differences in the number of sampling locations may also need to be accounted for when comparing the level of intraspecific genetic differentiation between the two species. *F*_ST_ values are likely to be overestimated when the number of sampling locations is limited (Chen et al., 2015). In this study, however, *P. herzensteini* was sampled at fewer locations but nevertheless exhibited lower *F*_ST_ values, suggesting that the observed difference in genetic differentiation between the two species is plausible. Third, we acknowledge that partial mtDNA sequence data have limited resolution compared to the complete mitogenome or nuclear genome data. Using multiple nucleotide markers could provide deeper insights into the factors influencing population genetic structure, offering higher resolution than partial mtDNA data alone.

### 4.2 Population genetic structure

With a focus on the intraspecific population genetic structure, geographical features and paleogeographic changes may have contributed to the highly differentiated population genetic structure of *P. yokohamae*. If ecological factors alone affect population genetic structures, a clear IBD pattern is expected because the dispersal of eggs and individuals is inversely proportional to geographical distance (Hellberg et al., 2002). The Japan Sea and Tohoku Pacific groups displayed this pattern; however, no significant IBD pattern was found in the entire dataset (Figure S4). Instead, some groups were highly genetically differentiated, with strong genetic barriers to gene flow between geographically adjacent sampling locations (Figures 4, 5 and S3b). For example, the Tokai group exhibited low genetic diversity and large genetic differences between adjacent sampling locations, primarily due to the presence of rare haplotypes. Similar patterns have also been reported in previous studies (Ikeda et al., 2010; Park et al., 1990). Given the locations of the strong genetic barriers and geographic characteristics of the Japanese Archipelago, deep troughs may act as barriers to gene flow (Figure 5). They possibly render the surrounding areas beyond the depth range suitable for *P. yokohamae*, resulting in habitat patchiness. The two major troughs, the Suruga and Sagami Troughs, correspond to the genetic break between the Kanto and Tokai groups. The Kamogawa Submarine Canyon is also consistent with the genetic barrier to gene flow between the Tohoku Pacific and Kanto groups. Although no clear genetic differences were observed around the Toyama Trough in this study, genetic differentiation across the Noto Peninsula has been reported in other species, including the Pacific cod *Gadus macrocephalus* Tilesius, 1810 and the dwarf eelpout *Petroschmidtia teraoi* (Katayama 1943) (Sakuma et al., 2020; Suda et al., 2017). For other genetically distinct groups (i.e. Kyushu, Tokai and Seto Inland Sea groups), paleogeographic changes likely influenced the present‐day population genetic structures (see Section 4.3).

In *P. herzensteini*, no significant genetic differentiation was detected among the sampling locations, consistent with the haplotype network and BAPS cluster composition in each location, both of which indicate substantial genetic mixture among individuals (Figures 2a and 3). Nevertheless, this does not rule out the possibility of subtle population genetic structure of this species. First, regional variations in spawning season and growth rate have been reported (Minami, 1995, 2008). For example, spawning time varies by nearly half a year within the Hokkaido region, ranging from March to April in Hakodate (southern Hokkaido) to July to August in Rausu (northeastern Hokkaido) (Minami, 1995). In Hokkaido, the following three regional groups have been recognized based on differences in the growth or infestation rates of copepod species (Nagasawa & Maruyama, 1987; Watanobe, 2003): Japan Sea growth group, Okhotsk Sea growth group and Abashiri Bay group. Second, fisheries data show that trends in catch and estimated biomass vary among regions (Chiba et al., 2024; Sato et al., 2024). Although differences in population dynamics among areas do not necessarily entail genetic differentiation, they can be indicators of population subdivision (Ovenden, 2013). Moreover, genomic studies have increasingly reported genetic differentiation associated with local adaptation in marine organisms, including migratory species, where extensive gene flow is maintained across the seascape (e.g. Barth et al., 2019; Diopere et al., 2018; Limborg et al., 2012). Genomic data analysis may provide new insights into the population genetic structure and local adaptation of *P. herzensteini* that cannot be captured using partial mtDNA data.

More comprehensive sampling potentially increases the resolution of population genetic structures, such as by identifying new genetic groups and re‐delineating existing groups or genetic barriers to gene flow. Because the sampled locations did not encompass the full distribution ranges of these species, undetected genetic differentiation may be present in unsampled regions. This is particularly likely for *P. yokohamae*, where finer‐scale population subdivision may be uncovered with more intensive sampling. Indeed, four regional populations with different spawning seasons have been reported around the Tsugaru Strait (Fujii, 2010; Ishino & Sano, 1996): Kikonai Bay, Tsugaru Peninsula, Mutsu Bay and Shimokita Peninsula groups. In addition to spatial sampling coverage, potential methodological constraints and temporal sources of variation also warrant consideration. For example, the genetic groups and barriers detected by SAMOVA and BARRIER may reflect the influence of sampling design (Dupanloup et al., 2002; Manni et al., 2004). Furthermore, population genetic compositions in marine organisms may change due to spatiotemporal variation in individual reproductive success and selection pressure (Liggins et al., 2020). Therefore, it would be desirable to reassess the population genetic structures of both species using samples from broader geographic coverage and different generations to evaluate the robustness of the inferred structures.

For *P. yokohamae*, this study focused only on individuals carrying *P. yokohamae* mtDNA haplotypes, although some individuals with *P. schrenki* haplotypes were identified primarily from the northern Japanese Archipelago (Table 1), as reported by Tsukagoshi et al. (2015). Compared to *P. yokohamae*, *P. schrenki* is distributed in more northern waters and occurs only in Hokkaido and Aomori (the northernmost prefecture of the Japanese mainland) in Japan (Nishiuchi, 1991). Given that haplotypes of *P. schrenki* have been detected outside the distribution range, introgression into *P. yokohamae* may have occurred through hybridization, followed by backcrossing, potentially influencing the present‐day population genetic structure of *P. yokohamae*. To investigate the hybridization and introgression of these species, analysis of nuclear DNA markers, rather than maternally inherited mtDNA, is essential.

### 4.3 Population demographic history

Sea‐level oscillations associated with the Pleistocene glacial–interglacial cycles led to remarkable paleogeographic changes in marine environments (Chappell, 1994; Lambeck et al., 2002). Particularly during the Last Glacial Maximum (LGM; approximately 29–19 kya), sea level was estimated to have been over 110 m lower than present day (Chappell, 1994). The Soya Strait was completely land‐bridged, while the Tsushima and Tsugaru Straits were likely constrained channels (Oba et al., 1991, Matsui et al., 1998; Figure 1). The Japan Sea was nearly isolated from the surrounding seas during the LGM, which was accompanied by a decline in salinity due to the excess input of freshwater from rivers (Oba et al., 1991; Tada, 1994). Such extreme environmental changes indeed have affected the population demographic histories and population genetic structures of many coastal fish species around the Japanese Archipelago (Matsui, 2022). Because both *P. herzensteini* and *P. yokohamae* inhabit coastal areas, most of their present‐day habitats were likely terrestrial during glacial periods, which would have greatly affected their population demographic histories.

Historical demographic analyses suggest that the paleogeographic changes had a greater impact on the demographic history and population divergence of *P. yokohamae* than those of *P. herzensteini*. From a paleogeographic perspective, *P. herzensteini* around the Japanese Archipelago was presumably temporarily isolated into three regions during the LGM: the Japan Sea, the Sea of Okhotsk and the Pacific Ocean. However, historical demographic analyses suggest that it has maintained a large population size over time with indication of population expansion and no genetic imprints of population subdivision or secondary contact (Figures 2a and 6a). These results imply that it could currently maintain sufficient gene flow across the seas despite temporary disruptions in migration caused by the past extreme geographic changes. In contrast, each genetic group of *P. yokohamae* exhibited distinctive inferred demographic histories. Based on patterns observed in haplotype networks, mismatch distributions and BSPs (Figure 6b–g), different demographic scenarios related to the paleogeographic changes are suggested, including the possibility of bottleneck events, establishment of refugial populations and secondary contact between genetically differentiated populations (see below). A comparative historical demographic study of coastal goby species around the Japanese Archipelago reported that those with lower dispersal potential in early life stages exhibited more pronounced phylogeographic structures, shaped by environmental changes associated with glacial–interglacial cycles (Hirase, 2022). This finding aligns with our results, suggesting that differences in early life histories may influence not only population genetic structure, but also demographic history.

With a focus on the demographic history of each genetic group of *P. yokohamae*, the Kyushu and Seto Inland Sea groups displayed the bottleneck‐shaped haplotype networks with star‐like branching patterns, multimodal mismatch distributions and recent population expansion in the BSPs (Figure 6b,g). Based on these results, several possibilities can be inferred, including a series of historical contractions and expansions over time, secondary contact among multiple refugial populations and post‐LGM population expansion. The present‐day habitats of these groups would have been repeatedly exposed during the glacial periods in the Late Pleistocene because they occur in shallow waters (i.e. mean depth of approximately 20 m in the Ariake Sea, 11 m in Hakata Bay and 38 m in the Seto Inland Sea; Figure 1). Such events may result in population size contractions, followed by rapid post‐LGM expansion. Alternatively, rising sea levels during interglacial periods may have facilitated recolonization and secondary contact among refugial populations, followed by admixture events that ultimately gave rise to the present‐day populations. The East China Sea and the Tsushima Strait are possible regions where refugial populations were established, as suggested by several phylogenetic studies on marine fish species (Hirase & Ikeda, 2014; Kokita & Nohara, 2011; Sakuma et al., 2019).

The Japan Sea and Tohoku Pacific groups represented the diffuse haplotype networks with star‐like branching patterns, unimodal mismatch distributions and population expansion in the BSPs (Figure 6c,d). These results suggest that they maintained large population sizes over time and experienced relatively recent population expansion. The estimated timing of the population expansion appears to correspond to interglacial periods in both groups in the BSPs, although the timings differ. Based on previous demographic studies, a large part of the coastal region along the Japan Sea is inferred to have become unsuitable for temperate coastal organisms during the LGM due to the low‐salinity environment (Matsui, 2022). Given the severe habitat conditions in the Japan Sea during the LGM, it is likely that the population size of the Japan Sea group increased after the LGM, which is consistent with the estimates from the BSP (Figure 6c). In contrast, the Tohoku Pacific group appears to have maintained a large population size over time, even during the LGM, suggesting that environmental conditions may have been less severe than those in the Japan Sea. Nevertheless, it is unlikely that the environmental conditions were uniformly favourable across the Pacific coast during the period. A relatively recent population expansion was suggested in the adjacent Kanto group (Figure 6e), implying that the substantial habitat loss (e.g. Tokyo Bay) caused by sea‐level decline during the LGM may have also affected demographic history along the Pacific coast.

The Tokai group exhibited a star‐like haplotype network, L‐shaped mismatch distribution and population history of only approximately 900 years in the BSP (Figure 6f). These historical demographic patterns suggest several possibilities, such as a recent population expansion, a selective sweep and a recent bottleneck event. Considering these results together with the low genetic diversity (Table 1), substantial genetic differentiation in this group (Figures 4 and S3b), significant deviation from neutrality (Table S3) and the restricted potential habitat area around this region during the LGM (Figure 1), the following demographic scenarios may explain the observed pattern: (1) the population experienced a severe bottleneck event during the LGM and a recent increase in population size; (2) the population either did not originally inhabit the region or became extinct, but migrants from neighbouring groups—possibly from the Kanto or Seto Inland Sea group—have recently colonized the area; and (3) the population underwent a strong recent selective sweep.

We acknowledge that inferences of population demographic history based on a single locus (i.e. mtDNA) may carry the risk of estimation errors (Kimura, 2013). For example, the timescale of the reconstructed demographic histories in BSPs can vary depending on the mutation rate (Grant, 2015). We applied the commonly used mutation rate for fish species; however, it may deviate from the species‐specific rate. Because the mutation rate generally varies among mtDNA regions and taxa (Rand, 1994; Zhuang et al., 2013), calibration using datable geological events or fossil records is recommended for accurate historical demographic inferences (Ho, 2008). For haplotype network analysis, it provides a useful overview of population demographic history, including signals of marine glacial refugia, secondary contact and recent population recolonization (Avise, 2000; Koizumi & Ikeda, 2013; Maggs et al., 2008). Nevertheless, the demographic histories inferred from haplotype networks may vary or be misinterpreted depending on the mutation rates of the mtDNA regions analysed (Yamamoto et al., 2024). Moreover, historical demographic analyses using mtDNA sequences alone have limited resolution, as they trace only maternal population histories. Indeed, multiple demographic scenarios are considered but cannot be resolved with mtDNA data, such as the Kyushu, Tokai and Seto Inland Sea groups in *P. yokohamae*. Further detailed demographic histories, including hybridization history between *P. yokohamae* and *P. schrenki*, can be inferred by analysing multiple nuclear loci.

### 4.4 Implications for fisheries management of both species

Establishing MUs that reflect underlying population genetic structure is essential for effective conservation and fisheries management (Hauser & Carvalho, 2008; Laikre et al., 2005; Palsbøll et al., 2007; Schwartz et al., 2007). For accurate stock assessments in exploited species, it is recommended that such units be defined through interdisciplinary integration of genetic, fishery‐dependent and ecological information (Cadrin et al., 2023). Simulation studies assessing the consequences of spatial mismatches between population genetic structures and MUs have shown that applying a single MU across genetically differentiated populations increases the risk of overfishing compared to managing them as independent MUs (Spies et al., 2015; Ying et al., 2011). This suggests that the spatial design of small‐scale MUs is a precautionary approach when information on population genetic structure is limited.

Stock assessment and management for *P. herzensteini* are currently being conducted for the northern Hokkaido stock and the Japan Sea stock in Japan (Chiba et al., 2024; Sato et al., 2024). No genetic differentiation was found among sampling locations of *P. herzensteini* in this study; however, fishery statistics and ecological studies have reported regional differences in population dynamics, growth rates and spawning seasons (see Section 4.2). For *P. yokohamae*, no comprehensive stock assessments and management have been conducted to date. In future resource management and stock assessments for this species, the six genetic groups identified in this study should be taken into account when defining MUs. In addition, the localized distribution of spawning grounds within individual bays or seas, and even smaller areas (Tanda et al., 2008), may contribute to genetic differentiation at spatial scales finer than those detected in this study, particularly around the Tsugaru Strait (Fujii, 2010; Ishino & Sano, 1996). Considering all these findings discussed herein, both species may need to be managed at smaller geographic scales, such as bays or seas, to mitigate the risk of overexploitation. Given the limited analytical resolution associated with using only partial mtDNA sequences in this study, and the possibility of hybridization with closely related species particularly in *P. yokohamae*, future efforts should examine MUs using nuclear DNA markers.

## AUTHOR CONTRIBUTIONS

Y.Y. contributed to investigation and writing – original draft. H.T., Y.K., S.K., Y.A., M. Ishii and T.I. contributed to resources and to reviewing and editing. M. Ikeda acquired funding and contributed to conceptualization. All authors critically reviewed and approved the final version of the manuscript.

## FUNDING INFORMATION

This study was funded by JSPS KAKENHI Grant Number JP19K06202.

## Supporting information


**TABLE S1.** Comparison of early life‐history traits between *Pseudopleuronectes herzensteini* and *P. yokohamae*.
**TABLE S2.** Nucleotide substitution models and MCMC settings used in Bayesian skyline plot analysis.
**TABLE S3.** Results of neutrality tests and mismatch distribution analyses.
**FIGURE S1.** Schematic diagram of the early life histories of *P. herzensteini* and *P. yokohamae*.
**FIGURE S2.** Neighbour‐joining tree of *P. yokohamae* and *P. schrenki* haplotypes based on control region sequences in mitochondrial DNA.
**FIGURE S3.** Heatmaps of pairwise *F*_ST_ values among sampling locations of *P. herzensteini* and *P. yokohamae*.
**FIGURE S4.** Relationships between geographical and genetic distances in P. *yokohamae*.

## Data Availability

The data that support the findings of this study are available in the GenBank/EMBL/DDBJ databases under accession numbers LC882785‐LC883537.
