# Supplementary material for: Contrasting population genetic structures of congeneric coastal flounders associated with different early life histories
Source: J Fish Biol. 2026 May 21;109(2):1141–59. doi: 10.1111/jfb.70461 (PMC13521706; doi:10.1111/jfb.70461)
Supplement: Supplementary file 1 [file JFB-109-1141-s001.doc]

**TABLE S1** Comparison of early life-history traits between *Pseudopleuronectes herzensteini* and *P. yokohamae*.

**Supporting information**

| Life-history traits | *P. herzensteini* | *P. yokohamae* |
| --- | --- | --- |
| Depth of spawning sites | 50–90 m (Tominaga et al., 1991) | 3.9–48 m (Minami, 1981; Nakagami et al., 2001; Takahashi, 2008; Tanda & Yamamoto, 2008) |
| Egg type | Pelagic eggs (Amaoka, 2016) | Adhesive demersal eggs (Amaoka, 2016) |
| Egg size | 0.86–0.95 mm (Ikeda, 1983) | 0.812–0.845 mm (Yusa, 1960) |
| Time to hatch | 6–7 days (Yamamoto et al., 1963; Aritaki, 2013) | 5–18 days (Yusa, 1972) |
| Size at hatchery | 3.02 mm TL (Aritaki & Seikai, 2004) | 3.46 mm TL (Fukuhara, 1988) |
| Migratory behavior and transportation of eggs or larvae to suitable nursery grounds | Produced eggs at bottom gradually float and the hatched larvae ontogenetically migrate to bottom (Imura et al., 2004). Egg and larval dispersal driven by ocean currents and coastal eddies has been suggested (Nakata, 1996; Suenaga et al., 1996). | Selective tidal-stream transport and wind-induced compensatory drift likely facilitate settlement at suitable sites (Takahashi et al., 1986; Nakagami et al., 2001). |
|  |  |  |
| Timing of settlement | Larvae shift to benthic life at the beginning of eye migration (Aritaki, 2013). | Larvae shift to benthic life at the beginning of eye migration (Fukuhara, 1988). |
| Size at settlement | 9.0 mm SL (Minami, 2001) | 8.2 mm SL (Minami, 2001) |
| Depth of settlement sites | 25–45 m (Aritaki & Yoseda, 1994) | Approx. 15 m (Minami, 1981; Nakagami et al., 2001) |
| Planktonic larval duration (hatching to settlement) | Approx. 40 days | Approx. 30 days |
| Experimental data | Water temperature 6 °C: 58 days, 9 °C: 47 days, 12 °C: 34 days, 15 °C: 25 days (Aritaki, 2013) | Water temperature 10 °C: 40 days, 14 °C: 30 days, 18 °C: 24 days (Mutsutani, 1988) |
| Data from wild populations | 41 (31–54) days (Joh & Wada, 2018) | 33–36 days (based on Joh et al., 2013 and Joh et al., 2005) |
| Metamorphosis duration | Approx. 6 days (Aritaki, 2013) | Approx. 9 days (Fukuhara, 1988) |
| Transportation or migration after settlement (juvenile stage) | Juveniles move slightly offshore to deeper waters after settlement, but then migrate little further (Fujii et al., 2008). | Juveniles actively migrate to inshore area at depths of 3–10 m using tidal currents (Takatsu, 2022). |

Abbreviations: TL, total length; SL, standard length.

**TABLE S2** Nucleotide substitution models for each mitochondrial DNA region and Markov chain Monte Carlo (MCMC) settings used in Bayesian skyline plot analysis.

| Population | Nucleotide substitution model | | MCMC generations |
| --- | --- | --- | --- |
|  | ND2 | Cyt *b* |  |
| *P. herzensteini* | TN93 | TN93 | 3.0 × 10^8^ |
| *P. yokohamae* |  |  |  |
| Kyushu group | HKY | HKY | 1.0 × 10^8^ |
| Japan Sea group | TN93 | TN93 | 1.0 × 10^8^ |
| Tohoku Pacific group | TN93 | TN93 | 2.0 × 10^8^ |
| Kanto group | HKY | HKY | 1.0 × 10^8^ |
| Tokai group | HKY | HKY | 2.5 × 10^8^ |
| Seto Inland Sea group | TN93 | HKY | 2.0 × 10^8^ |

Abbreviations: TN93, Tamura-Nei (Tamura & Nei, 1993); HKY, Hasegawa-Kishino-Yano (Hasegawa et al., 1985).

**TABLE S3** Results of neutrality tests and mismatch distribution analyses.

| Population | *D* | *F*_S_ | *R*_2_ | SSD | Hri |
| --- | --- | --- | --- | --- | --- |
| *P. herzensteini* | −2.1096^***^ | −24.1419^**^ | 0.0240^***^ | 0.0016 | 0.0024 |
| *P. yokohamae* |  |  |  |  |  |
| Kyushu group | −1.7680^*^ | −10.3264^**^ | 0.0445^**^ | 0.0056 | 0.0169 |
| Japan Sea group | −2.1084^***^ | −25.5243^***^ | 0.0230^***^ | 0.0026 | 0.0100 |
| Tohoku Pacific group | −1.9356^**^ | −24.8725^***^ | 0.0259^**^ | 0.0020 | 0.0062 |
| Kanto group | −1.4346^*^ | −6.3878^*^ | 0.0543^*^ | 0.0069 | 0.0144 |
| Tokai group | −2.0938^**^ | −15.3052^***^ | 0.0289^**^ | 0.0024 | 0.2107 |
| Seto Inland Sea group | −1.4488^*^ | −24.2033^***^ | 0.0433^*^ | 0.0033 | 0.0062 |

Abbreviations: *D*, Tajima’s *D*; *F*_S_, Fu’s *F*_S_; *R*_2_, Ramos-Onsins and Rozas’s *R*_2_; SSD, Sum of squared deviations; Hri, Harpending’s raggedness index.

^*^*p* < 0.05; ^**^*p* < 0.01; ^***^*p* < 0.001.

**FIGURE S1** Schematic diagram of the early life histories of coastal flounders *Pseudopleuronectes herzensteini* and *P. yokohamae*. Detailed ecological information is provided in Table S1. (a) Schematic representation of the differences in development during early life stages. Bars represent approximate duration of each life stage in wild populations. Arrows indicate planktonic duration during which eggs and/or larvae are dispersed by ocean currents. (b) Graphical representation of the differences in ecological characteristics and expected dispersal potential (shown by dotted arrows) in early life stages. The ontogenetic vertical migrations of eggs and/or larvae of *P. herzensteini* and *P. yokohamae* are depicted based on Imura et al. (2004) and Nakagami et al. (2001), respectively. Morphological illustrations of larvae of *P*. *herzensteini* and *P. yokohamae* are adapted with permission from Aritaki and Seikai (2004) and Fukuhara (1988), respectively.

**FIGURE S2** Neighbor-joining tree of *Pseudopleuronectes yokohamae* and *P. schrenki* haplotypes based on 332 bp of control region sequences in mitochondrial DNA. Black circle, haplotypes of *P. yokohamae* (*N* = 276); blue triangle, haplotypes of *P. schrenki* reported by Tsukagoshi et al. (2015) (*N* = 55); yellow square, haplotypes of *P. herzensteini* (*N* = 5); red circle, haplotypes of *P. schrenki* detected from morphologically *P. yokohamae* samples in this study (*N* = 14). The numbers on the nodes indicate 1000 bootstrap probabilities (values below 95% are omitted). Scale bar represents evolutionary distances based on the Tamura-Nei method (Tamura & Nei, 1993).

**FIGURE S3** Heatmaps of pairwise *F*_ST_ values among (a) the 6 sampling locations of *Pseudopleuronectes herzensteini* and (b) the 22 sampling locations (6 genetic groups) of *P. yokohamae*. See Table 1 for abbreviations of sampling locations. ^*^*p* < 0.05; ^**^significant differences following sequential Bonferroni correction (initial α = 0.00022).

**FIGURE S4** Pairwise relationships between geographical distance and genetic distance (*F*_ST_) for (a) all locations and (b) the Japan Sea and Tohoku Pacific groups of *Pseudopleuronectes yokohamae.* In (b), solid circles indicate the pairwise relationship within the Japan Sea group. The tendency line represents the fitted linear regression between the two variables.

**REFERENCES**

Amaoka, K. (2016). *Flatfishes of Japan (Citharidae, Paralichthyidae, Bothidae, Pleuronectidae, Poecilopsettidae, Samaridae)*. Tokai University Press. (in Japanese).

Aritaki, M. (2013). Studies on the morphological abnormality of Pleuronectidae: Focusing on the metamorphosis-related malformation in hatchery reared fish and its prevention. *Bulletin of Fisheries Research Agency*, *37*, 147–197. (in Japanese with English Abstract).

Aritaki, M., & Seikai, T. (2004). Temperature effects on early development and occurrence of metamorphosis-related morphological abnormalities in hatchery-reared brown sole *Pseudopleuronectes herzensteini*. *Aquaculture*, *240*, 517–530.

Aritaki, M., & Yoseda, K. (1994). The settlement and nursery ground of brown sole off the coast of Niigata Prefecture. *Nippon Suisan Gakkaishi*, *60*, 29–34. (in Japanese with English Abstract).

Fujii, T., Kajihara, N., & Iseki, T. (2008). Ecology of juvenile fishes revealed by the R/V Mizuho-maru surveys. *Major research findings of Japan Sea National Fisheries Research Institute*, *3*, 14–17. (in Japanese).

Fukuhara, O. (1988). Morphological and functional development of larval and juvenile *Limanda yokohamae* (Pisces: Pleuronectidae) reared in the laboratory. *Marine Biology*, *99*, 271–281.

Hasegawa, M., Kishino, H., & Yano, T. (1985). Dating of the human-ape splitting by a molecular clock of mitochondrial DNA. *Journal of Molecular Evolution*, *22*, 160–174.

Ikeda, T. (1983). Identification of eggs without oil droplets appearing in the coast of Wakasa Bay from winter to spring. *The Aquiculture*, *31*, 81–87. (in Japanese).

Imura, K., Takatsu, T., Nanjo, N., Kimura, O., & Takahashi, T. (2004). Spatial distribution of brown sole *Pseudopleuronectes herzensteini* eggs and larvae in Mutsu Bay. *Nippon Suisan Gakkaishi*, *70*, 39–47. (in Japanese with English Abstract).

Joh, M., Nakaya, M., Yoshida, N., & Takatsu, T. (2013). Interannual growth differences and growth-selective survival in larvae and juveniles of marbled sole *Pseudopleuronectes yokohamae*. *Marine Ecology Progress Series*, *494*, 267–279.

Joh, M., Takatsu, T., Nakaya, M., Higashitani, T., & Takahashi, T. (2005). Otolith microstructure and daily increment validation of marbled sole (*Pseudopleuronectes yokohamae*). *Marine Biology*, *147*, 59–69.

Joh, M., & Wada, A. (2018). Inter-annual and spatial difference in hatch date and settlement date distribution and planktonic larval duration in yellow striped flounder *Pseudopleuronectes herzensteini*. *Journal of Sea Research*, *137*, 26–34.

Minami, T. (1981). The early life history of a flounder *Limanda yokohamae*. *Bulletin of the Japanese Society of Scientific Fisheries*, *47*, 1411–1419. (in Japanese with English Abstract).

Minami, T. (2001). Metamorphosis and settlement of pleuronectid species. In T. Senda, T. Minami, & I. Kinoshita (Eds.), *Natural history of fish juveniles* (pp. 67–81). Hokkaido University Press. (in Japanese).

Minami, T. (2008). Ecology of flatfishes. (Review). In National Association for the Promotion of Productive Seas (Ed.), *Review of early life histories of major commercial fishes* (pp. 263–297). National Association for the Promotion of Productive Seas. (in Japanese).

Mutsutani, K. (1988). Growth and metamorphosis of the marbled sole larvae *Limanda yokohamae* (Günter) in culture. *The Aquiculture*, *36*, 27–32. (in Japanese).

Nakagami, M., Takatsu, T., Nakaya, M., & Takahashi, T. (2001). Spatial and temporal distribution of larval and juvenile marbled sole *Pleuronectes yokohamae* in Hakodate Bay. *Bulletin of the Japanese Society of Fisheries Oceanography*, *65*, 85–93. (in Japanese with English Abstract).

Nakata, H. (1996). Coastal fronts and eddies: their implications for egg and larval transport and survival processes. In Y. Watanabe, Y. Yamashita, & Y. Oozeki (Eds.), *Survival strategies in early life stages of marine resources* (pp. 227–241). CRC Press.

Suenaga, Y., Nakata, H., Fujihara, M., & Nagasawa, T. (1996). Numerical simulation of the wind effect on the transport of brown sole eggs and larvae in and around the Sado Strait. *Fisheries Engineering*, *32*, 219–228. (in Japanese with English Abstract).

Takahashi, K. (2008). Spawning grounds of the marbled flounder *Pleuronectes yokohamae* in Sendai Bay. *Tohoku Sokouo Kenkyu*, *28*, 13–16. (in Japanese).

Takahashi, K., Hoshiai, G., & Abe, H. (1986). Distribution and migration of *Limanda yokohamae* (Günter) larvae in Ishinomaki Bay and Mangoku Bay. *The Aquiculture*, *34*, 1–8. (in Japanese).

Takatsu, T. (2022). *Strong-year classes: Surviving strategies of flatfishes and cods*. Kaibundo Publishing. (in Japanese).

Tamura, K., & Nei, M. (1993). Estimation of the number of nucleotide substitutions in the control region of mitochondrial DNA in humans and chimpanzees. *Molecular Biology and Evolution*, *10*, 512–526.

Tanda, M., & Yamamoto, S. (2008). Ecology of larvae and juveniles of the marbled flounder *Pleuronectes yokohamae* spawning in sand and gravel areas. In M. Tanaka, M. Tagawa, & K. Nakayama (Eds.), *Fish juveniles: Exploring diverse physiology and ecology* (pp. 97–107). Seibutsukenkyusya. (in Japanese).

Tominaga, O., Nashida, K., Maeda, T., Takahashi, T., & Kato, K. (1991). Annual life cycle and distribution of adult righteye flounder *Pleuronectes herzensteini* in the coastal waters of northern Niigata prefecture. *Nippon Suisan Gakkaishi*, *57*, 2023–2031. (in Japanese with English Abstract).

Tsukagoshi, H., Takeda, K., Kariya, T., Ozaki, T., Takatsu, T., & Abe, S. (2015). Genetic variation and population structure of marbled sole *Pleuronectes yokohamae* and cresthead flounder *P. schrenki* in Japan inferred from mitochondrial DNA analysis. *Biochemical Systematics and Ecology*, *58*, 274–280.

Yamamoto, M., Sasaki, A., & Kuroshima, K. (1963). Yellow striped flounder *Limanda herzensteini* in Abashiri area. *Hokusuishi Geppo*, *20*, 293–302. (in Japanese).

Yusa, T. (1960). Eggs and larvae of flatfishes in the costal waters of Hokkaido IV. Embryonic development of mud dab *Limanda yokohamae* Günther. *Bulletin of Tohoku Regional Fisheries Research Laboratory*, *17*, 15–30.

Yusa, T. (1972). Ecology of coastal fish species mainly flatfishes and ocean development. *Bulletin of the Japanese Society of Fisheries Oceanography*, *20*, 68–75. (in Japanese).
